# Breast Cancer with Neoductgenesis: Histopathological Criteria and Its Correlation with Mammographic and Tumour Features

**DOI:** 10.1155/2014/581706

**Published:** 2014-10-08

**Authors:** Wenjing Zhou, Thomas Sollie, Tibor Tot, Sarah E. Pinder, Rose-Marie Amini, Carl Blomqvist, Marie-Louise Fjällskog, Gunilla Christensson, Shahin Abdsaleh, Fredrik Wärnberg

**Affiliations:** ^1^Department of Surgical Sciences, Uppsala University, 751 85 Uppsala, Sweden; ^2^Department of Pathology, Örebro University, 701 85 Örebro, Sweden; ^3^Department of Pathology, Falun Central Hospital, 791 82 Falun, Sweden; ^4^Department of Research Oncology, King's College London, London SE1 9RT, UK; ^5^Department of Immunology, Genetics and Pathology, Uppsala University, 751 85 Uppsala, Sweden; ^6^Department of Oncology, Helsinki University Central Hospital, 00029 Helsinki, Finland; ^7^Department of Radiology, Oncology and Radiation Science, Uppsala University, 751 85 Uppsala, Sweden; ^8^Department of Surgery, Falun Central Hospital, 791 82 Falun, Sweden; ^9^Department of Surgery, Uppsala Academic Hospital, 751 85 Uppsala, Sweden

## Abstract

*Introduction*. Breast cancer with mammographic casting type calcifications, high grade DCIS with an abnormal number of ducts, periductal desmoplastic reaction, lymphocyte infiltration, and tenascin-C (TN-C) overexpression has been proposed to represent a more aggressive form of breast cancer and has been denominated as breast cancer with neoductgenesis. We developed histopathological criteria for neoductgenesis in order to study reproducibility and correlation with other tumour markers. *Methods*. 74 cases of grades 2 and 3 DCIS, with or without an invasive component, were selected. A combined score of the degree(s) of concentration of ducts, lymphocyte infiltration, and periductal fibrosis was used to classify cases as showing neoductgenesis, or not. Diagnostic reproducibility, correlation with tumour markers, and mammographic features were studied. *Results*. Twenty-three of 74 cases were diagnosed with neoductgenesis. The kappa value between pathologists showed moderate reproducibility (0.50) (95% CI; 0.41–0.60). Neoductgenesis correlated significantly with malignant type microcalcifications and TN-C expression (*P* = 0.008 and 0.04) and with ER, PR, and HER2 status (*P* < 0.00001 for all three markers). *Conclusions*. We developed histological criteria for breast cancer with neoductgenesis. Neoductgenesis, by our applied histopathological definition was related to more aggressive tumour biology and malignant mammographic calcifications.

## 1. Introduction

The theory of neoductgenesis in breast cancer was proposed by László Tabár, radiologist at the Department of Mammography, Falun Central hospital, Sweden [1, 2]. When classifying the mammographic appearance of early stage breast cancers, Tabár and coworkers identified a subgroup with characteristic “casting type” calcifications with or without an associated tumour mass. The calcifications were present in large numbers and were unnaturally tightly packed, pointing in all directions, often filling an entire lobe. The corresponding histopathological picture was described as cancer-filled duct-like structures associated with periductal lymphocytic infiltration and a periductal desmoplastic reaction. Tabár et al. proposed that this duct-forming process represents a special type of neoplasia generating a large volume of neoplastic tissue but not fitting in any classical group of invasive or* in situ* breast cancer. This group also described how tenascin-C (TN-C) overexpression was detectable around the ducts bearing ductal carcinoma* in situ* of the breast (DCIS) in the cases with poor prognosis in their study [1], indicating an epithelial-stromal interaction similar to that occurring during the normal development of the duct system.

DCIS is often detected by breast screening as mammographic microcalcification without any clinical symptoms. Preoperative biopsies of cases with overtly malignant microcalcification on the mammogram often reveal high grade DCIS with or without an invasive component [3, 4]. To distinguish between pure DCIS, which has an excellent prognosis, and a potentially more aggressive type of breast cancer with neoductgenesis as proposed by Tabár et al. would be of great value. However, we need a more precise definition of the entity of “neoductgenesis” to be able to test the reproducibility and then it will be possible to apply these criteria to patient cohorts and to evaluate the correlation to prognosis.

In this study, we have defined histopathological criteria for neoductgenesis, based on the description by Tabár et al. In a selected cohort of women with intermediate or high grade DCIS with or without an invasive component we applied these criteria to define a group with neoductgenesis and a group without. Our aim was to evaluate whether the diagnosis of breast cancer with neoductgenesis could be made in a reproducible way and if so, to study the correlation between neoductgenesis and mammographic features and common immunohistochemical (IHC) markers including TN-C. This study was not designed to study prognosis.

## 2. Methods

### 2.1. Patients

Our purpose was to identify a cohort of women with a substantial number of cases with possible neoductgenesis and a control group with breast cancers of the same type, that is, cases with intermediate or high DCIS with or without an invasive component but with no signs of neoductgenesis. The inclusion of cases was based on the original histopathological report. The original diagnostic haematoxylin and eosin (H&E) slides were not reviewed in the process of creating the study cohort. We included primary tumours but also four recurrences.

Seventy-four women from two different sources were selected. (A) We prospectively collected tumour tissue from cases with suspicious mammographic calcifications between 2005 and 2006 at Uppsala Academic Hospital. Of 64 cases, 31 were eligible according to predetermined histopathological criteria. The remaining 33 cases were either benign lesions or pure invasive breast cancer and were therefore excluded. (B) As the prospective collection of cases only resulted in 31 lesions, we also collected cases that were already biobanked at the pathological departments in Falun and Uppsala. Based on the original histopathological reports, 11 cases diagnosed between 1996 and 2002 were selected from the biobank in Falun and 32 cases diagnosed between 1988 and 2004 from the biobank in Uppsala. The Uppsala cases had been included in an earlier study [5]. The mammografic apperance was not evaluated before inclusion in these later 43 cases. The study was approved by the ethics committee of Uppsala University, Sweden (Dnr 2005:118 and 2007:315).

### 2.2. Definition of Neoductgenesis

The definition of neoductgenesis by Tabár and Tot was as follows: “Neoductgenesis represents abnormal branching of the ducts within a breast lobe resulting in an unnaturally large number of duct-like structures per square unit. It is a typical feature of some high-grade DCIS and is regularly associated with signs of altered epithelial-stromal interaction, like periductal lymphocytic infiltration and remodelling of the specialized periductal stroma.” Thus, the histological criteria included for evaluation in this study were (1) concentration of duct-like structures and loss of normal ductal-lobular architecture, as seen at low power scanning microscopic magnification; (2) intense periductal lymphocytic infiltration; and (3) fibrosis-like thickening of the periductal stroma. Tabár and Tot also found periductal TN-C accumulation in the cases with neoductgenesis [2].

**Figure 1:**
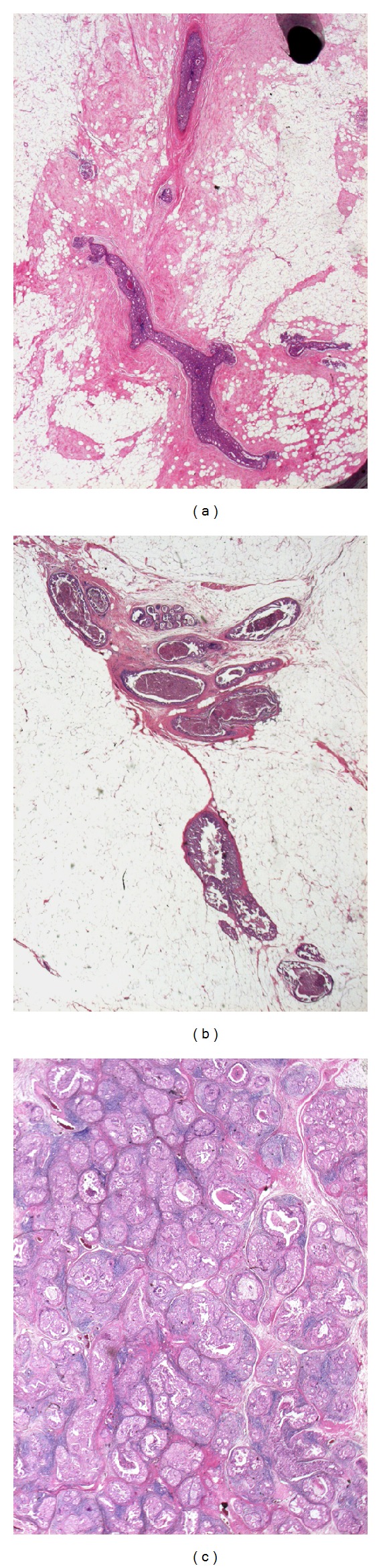
Concentration of duct-like structures and loss of normal ductal-lobular architecture. Hematoxylin-eosin stain; microscopic magnification 20×. (a) No concentration of duct-like structures; (b) focal concentration; (c) general concentration and loss of normal ductal-lobular architecture.

**Figure 2:**
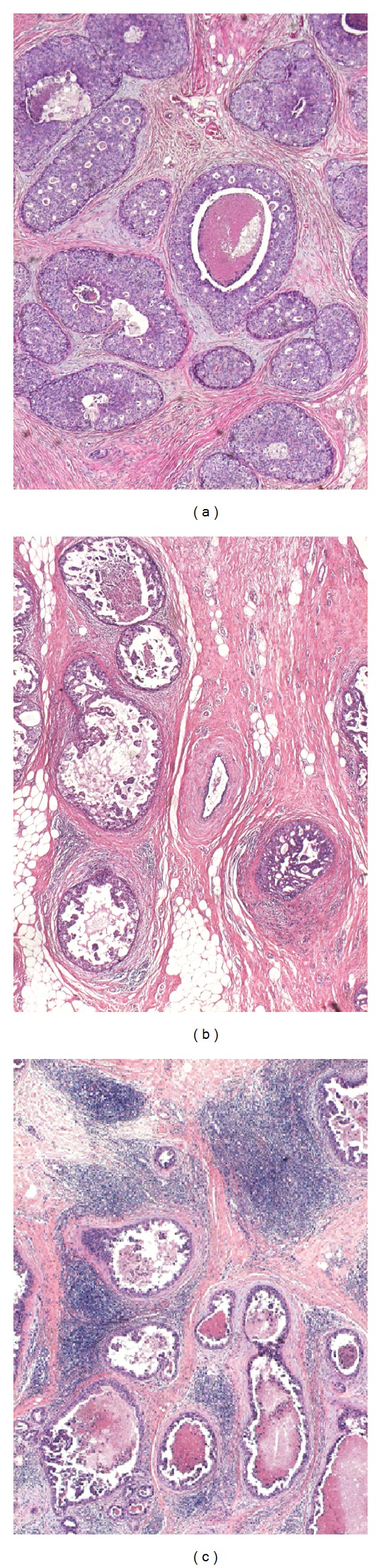
Lymphocytic infiltration. Hematoxylin-eosin stain; microscopic magnification 40×. (a) No periductal lymphocytic infiltration; (b) mild periductal lymphocytic infiltration; (c) intense periductal lymphocytic infiltration.

**Figure 3:**
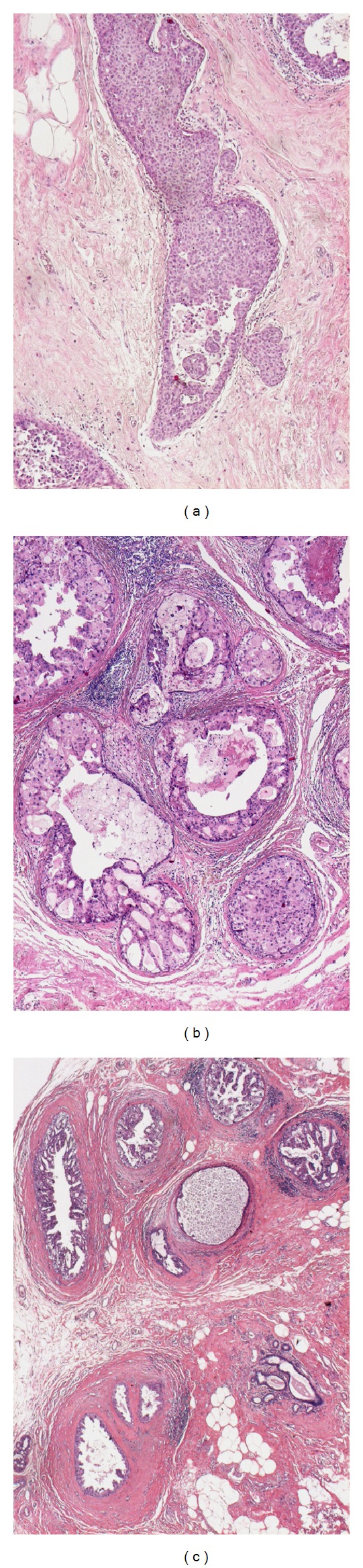
Fibrosis-like thickening of the periductal stroma. Hematoxylin-eosin stain; microscopic magnification 60×. (a) No periductal fibrosis; (b) little periductal fibrosis; (c) much periductal fibrosis.

In the present study, we quantified the degree of the concentration of ducts, lymphocyte infiltration, and periductal fibrosis as 0, 1, or 2, as per criteria for scoring presented in Table 1 and Figures 1, 2, and 3. A case with a combined score of 5 or 6 points was denominated as breast cancer with neoductgenesis. A case with a combined score of 0 to 4 points was denominated as a case without neoductgenesis. We did not include TN-C expression or presence of mammographic calcification in the definition of neoductgenesis in this study.

Up to five representative slides with tumour tissue from the original H&E slides from all cases were selected and then evaluated by four pathologists separately (T Tot, S Pinder, RM Amini, and H Nordgren) blinded to tumour biology and follow-up data. In some of the cases just one slide was available. Scoring of the concentration of duct-like structures, periductal lymphocytic infiltration, and fibrosis-like thickening of the periductal stroma was performed and assigned as 0, 1, or 2 by the different pathologists. In the analyses comparing cases with neoductgenesis or not we combined the scoring of all four pathologists and cases with a score of 17 to 24 points were denominated as breast cancer with neoductgenesis. Cases scoring 0 to 16 points were classified as not showing the features of neoductgenesis.

### 2.3. Mammographic Classification

Mammographic features were reclassified into six groups as described by Tabár et al. [1]: (1) a stellate lesion without associated calcifications; (2) a circular or oval mass without associated calcifications; (3) powdery calcifications with or without an associated tumour mass; (4) casting type calcifications with or without an associated tumour mass; (5) crushed stone-like (pleomorphic) calcifications with or without an associated tumour mass; and (6) others, that is, galactographic findings or nonspecific symmetric density.

Mammograms were reviewed by two independent radiologists (Tabár and Abdsaleh) blinded to tumour biology and follow-up. Based on the mammographic classification, all were categorized as being with or without malignant calcification (4 and 5 versus 1, 2, 3, and 6).

### 2.4. Tissue Microarray (TMA) and IHC

TMA blocks were constructed using archival formalin-fixed, paraffin-embedded specimens. H&E sections were reevaluated and appropriate tumour areas were selected. Two cores from the invasive and the* in situ* components, respectively, with a diameter of 1.0 mm were mounted into the recipient TMA block using a manual arraying device (MTA-1, Beecher Inc., WI, USA). The concordance of IHC staining between biopsies on TMA-slides and original whole section slides from the same DCIS lesion has previously been evaluated [6, 7]. We performed IHC for estrogen and progesterone receptors (ER, PR), HER2, Ki67, and TN-C on 3.5 *μ*m paraffin sections cut from the TMAs. TMA slides were immunostained in the Ventana automated Immunohistochemistry System (Ventana Benchmark XT and Ultra) using Ventana UltraView DAB (760-500). IHC was conducted according to established protocols. Appropriate positive and negative controls were included in all staining runs. The primary antibody tenascin-C DAKO, clone TN2, dilution 1 : 50, was used.

**Figure 4:**
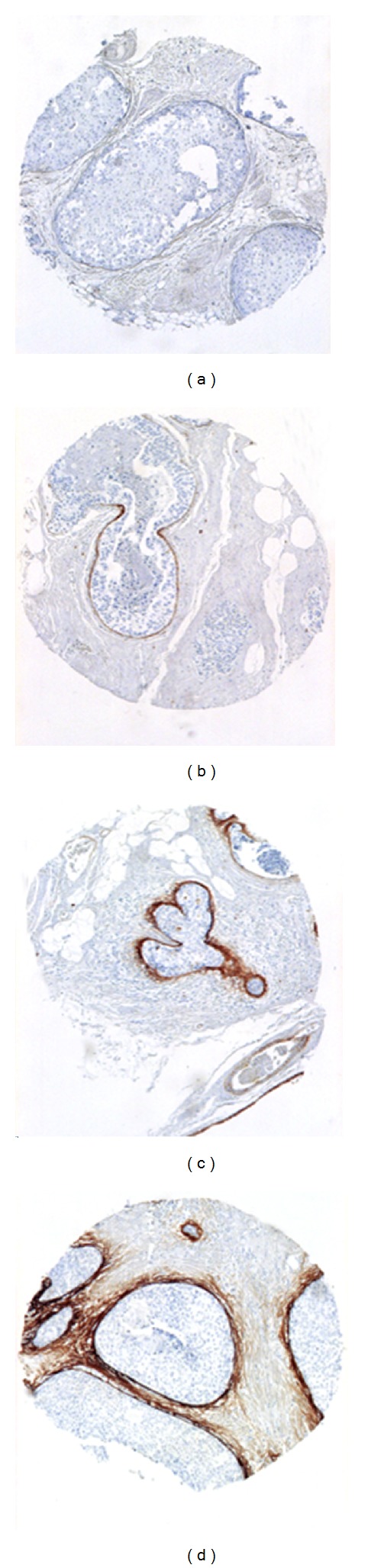
Tenascin-C scoring. Tenascin-C immunostaining; magnification 40×. TMA slide details with tenascin-C expression 0 (a), 1+ (b), 2+ (c), and 3+ (d).

Tumours that showed nuclear staining in 10% or more of the tumour cell nuclei were considered ER and/or PR positive. This is still used as the clinical cut-off for invasive breast cancer in Sweden. Using the HerceptTest classification system, tumours were considered HER2 positive if the score was 3+ and negative if the score was 0–2+. Ki67 stains were considered high if staining was seen in more than 20% of tumour nuclei. The scoring was done by one observer (T Sollie). The staining intensity of TN-C was assessed in the periductal area and judged as negative/normal = 0; weak = 1; moderate = 2; and intense = 3, as presented in Figure 4. TN-C staining was scored by one pathologist (T Tot) and cases with moderate or intense staining (2-3) were considered TN-C positive.

If only one core contained enough tumour tissue, this was used for classification but at least 200 cells had to be evaluable. In 51 of the cases, we had sufficient tissue for TMA construction. IHC data for the remaining 23 cases were collected from TMAs used in an earlier study or from original slides if available [8]. We scored the IHC in the DCIS component in the mixed cases primarily, but in two cases insufficient tumour material was retrieved from the DCIS component and we used the invasive cancer component instead. We have earlier shown that the ER and HER2 expression in the DCIS and invasive components of mixed cases correlate significantly [9].

### 2.5. Statistical Analysis

Fisher's exact tests were used to compare the distribution of clinical and histopathological characteristics of the primary DCIS in those with and those without neoductgenesis. Pathological scoring data were analyzed to test the consistency of four independent pathologists using the Fleiss's Kappa from rating scores. The interpretation of the Kappa results followed Shrout's instructions [10, 11].

## 3. Results

Baseline characteristics for the 74 cases, 32 with pure DCIS and 42 with DCIS and an invasive component, are shown in Table 2. ER and PR status were considered positive in 71.6% and 56.7% of all cases, respectively. HER2 overexpression and high proliferation by Ki67 were seen in 39.2% and 29.7%, respectively. Of the 74 cases, 48 showed malignant calcifications on the mammogram, according to one or both of the breast radiologists. The kappa value for the agreement on malignant calcifications versus not between the two radiologists was 0.77 (95% CI; 0.59–0.94). Of the 48 cases with malignant calcifications, 15 had casting type calcifications and 19 had crushed stone-like calcifications, classified as such by both observers. Six cases were classified as casting type by one radiologist and crushed stone-like calcifications by the other radiologist, and another six cases were classified as malignant calcifications by one reader but not by the other reader. In two cases, mammograms were not available for review and data was retrieved from the original report. In 40 of 51 cases successfully stained for TN-C, staining was classified as high (2-3 points) and the proportion of stained cases was similar among pure DCIS and mixed lesions (50.0% and 57.1%, resp.).

An increased concentration of ducts (1 or 2 points) was seen in 77.0% of the pure DCIS and in 69.0% of the mixed cases. Lymphocyte infiltration (1 or 2 points) was found in 59.5% of the pure DCIS and in 69.0% of the mixed cases. Mild or intense periductal fibrosis was seen in 90.6% of the pure DCIS and in 88.3% of the mixed cases. The correlation between each of these three histopathological features and common tumour markers are presented in Table 3.

Using our definition of neoductgenesis based on the three histopathological characteristics together, 23 cases were defined as breast cancer with neoductgenesis and 51 as breast cancer without neoductgenesis. The kappa value for the diagnosis of neoductgenesis versus not (5-6 points versus 0–4 points) was 0.50 (95% CI; 0.41–0.60) between the four pathologists. If we used the histopathological criteria combined with the mammographic criteria of casting type calcifications and with an increased TN-C expression, 14 cases were defined as showing neoductgenesis, as three cases of the 23 showing histopathological signs of neoductgenesis did not show casting type malignant calcifications, one was TN-C low, and another five cases had missing TN-C data.

The correlation between tumours with and without histopathological neoductgenesis and patient and other tumour markers is presented in Table 4. Age at diagnosis did not differ between women with a breast cancer with features of neoductgenesis and those without. Cases with neoductgenesis showed malignant calcifications on the mammograms significantly more often than cases without neoductgenesis (87.0% versus 54.9%, *P* = 0.008). Among the 23 cases with neoductgenesis, mammographic calcifications were classified as casting type by both breast radiologists in ten cases and crushed stone-like in six cases. Also, 23 of the 51 cases with no histopathological signs of neoductgenesis had malignant calcifications on the mammograms. Tumours with neoductgenesis were more often of cytonuclear grade 3, (82.6% versus 56.9%, *P* = 0.03). Neoductgenesis was also statistically significantly correlated with ER negativity (63.6% versus 8.2%, *P* < 0.00001), PR negativity (81.8 versus 22.4%, *P* < 0.00001), and HER2 positivity (90.5% versus 20.8%, *P* < 0.00001), compared with cases showing no signs of neoductgenesis. Eighteen cases were classified as HER2 2+ by IHC. The correlation between HER2 positivity and neoductgenesis was still statistically significant if these HER2 2+ cases were considered HER2 positive or if they were excluded from the analysis. High proliferation was also seen more often in cases with neoductgenesis, but this was only of borderline statistical significance (47.6% versus 25.0%, *P* = 0.06). TN-C staining was performed in 51 of the cases. Seventeen of the 18 cases (94.4%) with neoductgenesis showed an overexpression of TN-C and 23 of the 33 (69.7%) cases without neoductgenesis (*P* = 0.04).

## 4. Discussion

A new entity of breast cancer has been proposed: breast cancer with neoductgenesis, that is, a cancer often described as “DCIS” which in reality may behave as a duct forming invasive carcinoma. The original description was based on a combination of mammographic casting type calcifications, histopathological features, and TN-C expression. We defined and quantified the histopathological criteria for the diagnosis and by using these criteria the diagnosis of breast cancer with neoductgenesis could be made with moderate reproducibility. In this study, we only used these histopathological criteria to define neoductgenesis and we excluded mammographic features and TN-C expression. Instead we evaluated their correlation with the histopathological criteria. We also found that neoductgenesis according to our definition correlated highly with more aggressive tumour biology. This is potentially valuable in clinical management. As a group, women with a pure DCIS have an excellent prognosis. If the histopathological diagnosis of neoductgenesis confers, or indicates, a poorer prognosis, it would be of value for treatment decisions, such as whether to perform sentinel lymph node biopsy, especially if this diagnosis could be made on preoperative core needle biopsy of patients with DCIS.

The kappa value for the correlation between the four pathologists was 0.50. This is a moderate agreement according to Landis and Koch [12]. Further work is indicated to tighten the criteria, particularly in relation to any prognostic value; we believe that larger studies with clinical follow-up data are indicated. One potential explanation of this Kappa value could be that in many cases only one or two sections from each lesion were available for the pathologists. Some of the lesions were small and we had problems finding representative slides for all cases. The correlation should be better in a clinical setting where more representative tumour tissue will be available and when histological large sections can be used [13]. We look at this study as a first step trying to define neoductgenesis and we are well aware that the system may have to be improved.

In addition, the number of cases in this study was relatively low and they were selected from different sources and time periods and, furthermore, both pure DCIS and DCIS with an invasive component and recurrences were included. Also, different treatments were given. This makes any study of the relation to prognosis impossible. Instead, our aim was to create a study cohort enriched with cases of possible neoductgenesis and a comparable group with similar tumour characteristics except for neoductgenesis. We believe we managed to do this, but the next step will be a larger study in a less selected patient cohort where we also can relate our histopathological criteria to prognosis. An indication of that the entity of neoductgenesis might have a poorer prognosis was published on a consecutive material of 108 DCIS cases, reported earlier by Tot [13].

We also wish to study the biology behind the proposed entity of neoductgenesis. We do not have any evidence of the actual formation of new ducts but this has been proposed as the mechanism. Actually, of the features included in our definition of neoductgenesis in this study the presence of “concentration of duct-like structures and loss of normal ductal-lobular architecture” showed the weakest correlation with mammographic calcification and histological features of aggressiveness.

All three histopathological features: concentration of duct-like structures and loss of normal ductal-lobular architecture, intense periductal lymphocytic infiltration, and fibrosis-like thickening of the periductal stroma, correlated with cytonuclear grade and ER, PR, and HER2 expression to some degree. In a report from 2009 [14] by Chivukula et al. on preoperative core needle biopsies, regressive changes in high grade DCIS including periductal fibrosis and inflammation correlated with invasive cancer in the surgical specimen and axillary node metastasis. However, no correlation was studied between these regressive changes and other tumour markers, concentration of ducts, or mammographic features. In our study, lymphocytic infiltration and periductal fibrosis showed the strongest association with the histological features of aggressiveness and mammographic calcifications, while this association was weaker for concentration of duct-like structures. Interestingly, a strong association between chronic inflammation and cytonuclear grade as well as risk of local invasive recurrence in DCIS has recently been shown [15]. Furthermore, combining the three histopathological features, as in cases with neoductgenesis, resulted in a highly statistically significant association with the IHC markers.

The association with HER2 expression was especially noteworthy. The lesions that were not classified as showing neoductgenesis were HER2 positive in only 21% of the cases, while breast cancers with neoductgenesis were HER2 positive in approximately 90%. Neoductgenesis may thus be related to the HER2 positive molecular subgroup (HER2+/ER−/PR−) [16] and it would be interesting to study the gene expression of these lesions and also, data on HER2 amplification would strengthen the analyses. In invasive breast cancer the HER2 positive subgroup has been shown to be related to poor prognosis [17–19]. However, this is not very well studied in pure DCIS; we have seen indications of this being true also for* in situ* lesions [8].

It could be argued that the proposed entity of DCIS with neoductgenesis is similar to high grade, HER2 positive DCIS. Among the 32 pure DCIS cases in this study, nine were classified as DCIS with neoductgenesis and all the nine cases were high grade and seven out of eight cases with HER2 data were HER2 positive. On the other hand, six of the 13 high grade, HER2 positive DCIS were not classified as DCIS with neoductgenesis by the pathologists. Of all 74 cases, 28 were high grade and were HER2 positive. Of these 28, 10 were not classified as breast cancer with neoductgenesis.

In a clinical setting the mammographic picture may suggest before surgery whether the patient has a lesion with features of neoductgenesis. A preoperative core needle biopsy is typically performed for diagnosis; the usefulness of adapting the histopathological criteria described in the assessment of large needle biopsies has to be evaluated if there are implications for choice of surgical treatment. Again, the criteria for diagnosis, even if proven of value and reproducibility in whole sections, may have to be adapted for application to core biopsy samples.

In this study we did not include the mammographic features in the definition of neoductgenesis. Rather, we studied the correlation between the histopathology and the mammographic features and report a statistically significant correlation between histopathological neoductgenesis and malignant calcifications (casting type and/or crushed stone-like calcifications). However, many of the cases with no signs of neoductgenesis also presented with malignant microcalcifications on the mammograms.

TN-C is an extracellular glycoprotein involved in tissue interactions during embryogenesis (e.g., ductgenesis in the normal breast), wound healing, inflammation, and malignancy. TN-C is expressed by normal mesenchymal and epithelial cells as well as by some cancer cells. The characteristics of TN-C include antiadhesion effects and growth promotion by increasing the mitogenic effect of fibroblast growth factor and are a prerequisite for epidermal growth factor-induced proliferation [20, 21]. This suggests an important role for TN-C in tumour-matrix interactions, potentially regulating tumour proliferation, invasion, and metastasis. Loss of epithelial morphology and gain of mesenchymal characteristics may be seen in cancer cells in tumour progression [20]. TN-C has also been regarded as a potential marker for microinvasion in DCIS by some authors [22–25]. In a study by Jahkola et al., the prevalence of periductal TN-C staining in DCIS correlated with high grade, high proliferation, and PR negativity as well as microinvasion [20]. The reason not to include TN-C in our definition of neoductgenesis in this study was partly based pragmatically on the fact that we did not succeed with the assessment of expression of TN-C in more than 51 of the 74 cases. Subsequently, almost all cases showing neoductgenesis stained positively for TN-C (17/18) but, in parallel with malignant calcifications, nearly 70% of the cases without neoductgenesis also showed overexpression of TN-C in this highly selected study. We selected about half of the cases based on mammographic appearance and we also only included cases of intermediate or high grade DCIS. Despite this bias, we found a statistically significant correlation between our definition of neoductgenesis and TN-C expression. This also requires further assessment in larger series.

In conclusion, we have developed histological criteria for a proposed new entity of breast cancer: breast cancer with neoductgenesis. Neoductgenesis, by our definition, was related to more aggressive tumour biology, especially HER2 positivity and to mammographic calcifications. The number of cases is relatively small, there is case selection bias, and the results require validation. The biology behind the histopathological features and its potential relation to prognosis also needs to be further studied.

## Figures and Tables

**Table 1:** Criteria for scoring the concentration of duct-like structures, lymphocytic infiltration, and periductal fibrosis. The classification of “neoductgenesis” was based on these histopathological criteria. See Figures 1–3.

	Scoring	
(1) Concentration of duct-like structures and loss of normal ductal-lobular architecture	0	No concentration of duct-like structures and no loss of normal ductal-lobular architecture
	1	Focal concentration of duct-like structures and focal loss of normal ductal-lobular architecture
	2	General concentration of duct-like structures and loss of normal ductal-lobular architecture

(2) Lymphocytic infiltration	0	No periductal lymphocytic infiltration
	1	Mild periductal lymphocytic infiltration
	2	Intense periductal lymphocytic infiltration

(3) Fibrosis-like thickening of the periductal stroma	0	No fibrosis-like thickening of the periductal stroma
	1	Little fibrosis-like thickening of the periductal stroma
	2	Much fibrosis-like thickening of the periductal stroma

Neoductgenesis	Yes	5-6 points
	No	0–4 points

**Table 2:** Baseline characteristics in 74 women with DCIS with or without an invasive component.

Characteristics	All cases (*n* = 74)	Pure DCIS (*n* = 32)	DCIS with an invasive component (*n* = 42)
	Number (%)	Number (%)	Number (%)
Age at diagnosis			
≤55 years	29 (39.2)	14 (43.8)	15 (35.7)
>55 years	45 (60.8)	18 (56.2)	27 (64.3)
Mammographic casting type or crushed stone-like calcifications			
Yes	48 (64.9)	23 (71.9)	25 (59.5)
No	26 (31.1)	9 (28.1)	17 (40.5)
Cytonuclear grade			
2	26 (35.1)	7 (21.9)	19 (45.2)
3	48 (64.9)	25 (78.1)	23 (54.8)
ER			
Positive	53 (71.6)	22 (68.8)	31 (73.8)
Negative	18 (24.3)	7 (21.9)	11 (26.2)
Missing	3 (4.1)	3 (9.3)	0
PR			
Positive	42 (56.7)	18 (56.2)	23 (57.1)
Negative	29 (39.2)	11 (34.4)	16 (47.9)
Missing	3 (4.1)	3 (9.4)	0
HER2			
Positive	29 (39.2)	13 (40.6)	16 (38.1)
Negative	40 (54.1)	15 (46.9)	25 (59.5)
Missing	5 (6.8)	4 (12.5)	1 (2.4)
Ki67			
High	22 (29.7)	7 (21.9)	15 (35.7)
Low	47 (63.5)	21 (65.6)	26 (61.9)
Missing	5 (6.8)	4 (12.5)	1 (2.4)
Tenascin-C			
Positive	40 (54.0)	16 (50.0)	24 (57.1)
Negative	11 (14.9)	5 (15.6)	6 (14.3)
Missing	23 (31.1)	11 (34.4)	12 (28.6)
Concentration of duct-like structures∗			
No	17 (23.0)	4 (12.5)	13 (31.0)
Focal	33 (44.6)	15 (46.9)	18 (42.8)
General	24 (32.4)	13 (40.6)	11 (26.2)
Lymphocytic infiltration∗			
No	26 (35.1)	13 (40.6)	13 (31.0)
Mild	29 (39.2)	12 (37.5)	17 (40.5)
Intense	19 (25.7)	7 (21.9)	12 (28.5)
Fibrosis-like thickening of the periductal stroma∗			
No	8 (10.8)	3 (9.4)	5 (11.9)
Mild	52 (70.3)	24 (75.0)	28 (66.7)
Intense	14 (18.9)	5 (15.6)	9 (21.4)
Neoductgenesis∗∗			
Yes, 17–24	23 (31.1)	9 (28.1)	14 (33.3)
No, 0–16	51 (68.9)	23 (71.9)	28 (66.7)

∗The value is based on the average score among the four pathologists.

∗∗The value is based on the combined scores among the four pathologists.

**Table 3:** Correlation between the three different histopathological features included in the definition of breast cancer with neoductgenesis in 74 cases with intermediate (grade 2) or high (grade 3) DCIS grade, with or without an invasive component.

Characteristics	Nuclear grade 3		Malignant calcifications		ER+		PR+		HER2+		Ki67 high		Tenascin-C+	
	Number (%)	*P* value	Number (%)	*P* value	Number (%)	*P* value	Number (%)	*P* value	Number (%)	*P* value	Number (%)	*P* value	Number (%)	*P* value
Concentration of duct-like structures∗														
No (*n* = 17)	8 (47.1)	0.18	7 (41.2)	0.07	14 (87.5)	0.01	9 (56.2)	0.2	6 (37.5)	0.04	6 (37.5)	0.6	7 (77.8)	0.45
Focal (*n* = 33)	22 (66.7)		24 (72.7)		28 (84.8)		23 (69.7)		9 (29.0)		8 (25.8)		18 (72.0)	
General (*n* = 24)	18 (75.0)		17 (70.8)		11 (50.0)		10 (45.5)		14 (63.6)		8 (36.4)		15 (88.2)	
Lymphocytic infiltration∗														
No (*n* = 26)	13 (50.0)	0.02	13 (50.0)	0.06	23 (92.0)	0.001	22 (88.0)	0.0002	1 (4.2)	<0.00001	6 (25.0)	0.46	11 (78.6)	0.19
Mild (*n* = 29)	18 (62.1)		19 (65.5)		23 (79.3)		15 (51.7)		14 (48.3)		9 (31.0)		15 (68.2)	
Intense (*n* = 19)	17 (89.5)		16 (84.2)		7 (41.1)		5 (29.4)		14 (87.5)		7 (43.8)		14 (93.3)	
Fibrosis-like thickening of the periductal stroma∗														
No (*n* = 8)	4 (50.0)	0.16	5 (62.5)	0.04	6 (85.7)	<0.001	6 (85.7)	0.04	1 (14.3)	0.0001	2 (28.6)	0.20	3 (75.0)	0.70
Mild (*n* = 52)	32 (61.5)		30 (57.7)		43 (84.3)		32 (62.7)		17 (34.7)		13 (26.5)		29 (76.3)	
Intense (*n* = 14)	12 (85.7)		13 (92.9)		4 (7.7)		4 (30.8)		12 (92.3)		7 (53.8)		8 (88.9)	

∗The scores are based on the mean value between the four different pathologists.

**Table 4:** The correlation between neoductgenesis and patient and tumour characteristics in 74 cases with intermediate (grade 2) or high (grade 3) DCIS with or without an invasive component.

	Neoductgenesis∗		*P* value
	Yes (*n* = 23) Number (%)	No (*n* = 51) Number (%)	
Age at diagnosis (*n* = 74)			
≤55 years	10 (43.5)	19 (37.3)	0.61
>55 years	13 (56.5)	32 (62.7)	
Mammographic casting type or crushed stone-like calcifications (*n* = 74)			
Yes	20 (87.0)	28 (54.9)	0.008
No	3 (13.0)	23 (45.1)	
DCIS with or without an invasive component (*n* = 74)			
DCIS	9 (39.1)	23 (45.1)	0.63
DCIS + invasive	14 (60.9)	28 (54.9)	
Cytonuclear grade (*n* = 74)			
2	4 (17.4)	22 (43.1)	0.03
3	19 (82.6)	29 (56.9)	
ER (*n* = 71)			
Positive	8 (36.4)	45 (91.8)	<0.001
Negative	14 (63.6)	4 (8.2)	
PR (*n* = 71)			
Positive	4 (18.2)	38 (77.6)	<0.001
Negative	18 (81.8)	11 (22.4)	
HER2 (*n* = 69)			
Positive	19 (90.5)	10 (20.8)	<0.001
Negative	2 (9.5)	38 (79.2)	
Ki67 (*n* = 69)			
High	10 (47.6)	12 (25.0)	0.06
Low	11 (52.4)	36 (75.0)	
Tenascin-C (*n* = 51)			
Positive	17 (94.4)	23 (69.7)	0.04
Negative	1 (5.6)	10 (30.3)	

∗Neoductgenesis was defined as a total score of 17–24 between four pathologists, combining the scores for concentration of ducts (0–2), lymphocytic infiltration (0–2), and periductal fibrosis (0–2) for each pathologist.

## Abbreviations

DCIS: Ductal breast carcinoma* in situ*
TN-C: Tenascin-C
ER: Estrogen receptor
PR: Progesterone receptor
HER2: Human epidermal growth factor receptor 2
TMA: Tissue microarray
IHC: Immunohistochemistry
CI: Confidence interval.
